# Willingness-to-pay for long-lasting insecticide-treated bed nets: a discrete choice experiment with real payment in Ghana

**DOI:** 10.1186/s12936-019-3082-6

**Published:** 2020-01-13

**Authors:** Y. Natalia Alfonso, Matthew Lynch, Elorm Mensah, Danielle Piccinini, David Bishai

**Affiliations:** 1 Johns Hopkins Bloomberg School of Public Health, 615 N. Wolfe Street, Baltimore, MD 21205 USA; 2 Johns Hopkins Center for Communication Programs, 111 Market Place, Suite 310, Baltimore, MD 21202 USA; 3 URIKA Research, Konadu Office Plaza, 1st Floor, Suite 2, Community 4, Tema, Ghana

**Keywords:** Malaria, Long-lasting insecticide nets, Commercial private markets, Discrete choice experiment, Willingness-to-pay, Middle-income, Ghana

## Abstract

**Background:**

Expanding access to long-lasting insecticidal nets (LLINs) is difficult if one is limited to government and donor financial resources. Private commercial markets could play a larger role in the continuous distribution of LLINs by offering differentiated LLINs to middle-class Ghanaians. This population segment has disposable income and may be willing to pay for LLINs that meet their preferences. Measuring the willingness-to-pay (WTP) for LLINs with specialty features that appeal to middle-class Ghanaians could help malaria control programmes understand what is the potential for private markets to work alongside fully subsidized LLIN distribution channels to assist in spreading this commodity.

**Methods:**

This study conducted a discrete choice experiment (DCE) including a real payment choice among a representative sample of 628 middle-income households living in Ashanti, Greater Accra, and Western regions in Ghana. The DCE presented 18 paired combinations of LLIN features and various prices. Respondents indicated which LLIN of each pair they preferred and whether they would purchase it. To validate stated willingness-to-pay, each participant was given a cash payment of $14.30 (GHS 65) that they could either keep or immediately spend on one of the LLIN products.

**Results:**

The households’ average probability of purchasing a LLIN with specialty features was 43.8% (S.D. 0.07) and WTP was $7.48 (GHS34.0). The preferred LLIN features were conical or rectangular one-point-hang shape, queen size, and zipper entry. The average WTP for a LLIN with all the preferred features was $18.48 (GHS 84). In a scenario with the private LLIN market, the public sector outlay could be reduced by 39% and private LLIN sales would generate $8.1 million ($311 per every 100 households) in revenue in the study area that would support jobs for Ghanaian retailers, distributors, and importers of LLINs.

**Conclusion:**

Results support a scenario in which commercial markets for LLINs could play a significant role in improving access to LLINs for middle-income Ghanaians. Manufacturers interested could offer LLIN designs with features that are most highly valued among middle-income households in Ghana and maintain a retail price that could yield sufficient economic returns.

## Background

One of the most cost-effective strategies for reducing the global malaria burden is sleeping under long-lasting insecticidal bed nets (LLINs). It is estimated that LLINs offer a cost of $27 per disability-adjusted-life-year (DALY) averted [1, 2]. They are effective even in areas with mosquito resistance to insecticides [3]. Between 2010 and 2016, the proportion of people at risk of malaria in Africa sleeping under an insecticide-treated net (ITN), including LLINs, increased from 30 to 54% [4]. The 2016–2030 goal of the Malaria Global Technical Strategy (GTS) and the World Health Organization (WHO) is to achieve and maintain universal coverage with LLINs, specifically one net for every two persons at risk of malaria. Multiple strategies will be required to grow more coverage including mass free net distribution campaigns and the growth of new and under-utilized channels, such as commercial sector channels [5].

Expanding access to malaria control measures is difficult given the many demands on limited -government health budgets [6]. A strategy that has gained traction but needs further research is expanding the role private commercial markets could play in the distribution of LLINs. The essential principle is focusing scarce government resources on offering protection to those who cannot afford commercially sold products, but to allow the emergence of an upscale market to take some of the financial pressure off of the government. This pressure relief system cannot take root unless commercial firms choose to enter markets where they are competing against a flood of free bed net products. Commercial private sector sales can be an important source for supplying LLINs to non-poor households willing-to-pay for a LLIN. The commercial sector can also serve as a backstop for poor households in the event that public sector funding and channels cannot supply enough nets to increase coverage or replace worn-out nets [2].

Prior to the era of publicly-funded mass distribution campaigns for LLINs, a commercial market for LLINs existed in several African countries. While mass campaigns rapidly increased ownership of LLINs bringing major benefits to millions of families, it came at a cost to the commercial market, which has diminished due to a lack of incentives for the private sector given users’ dependency on donor-provided free nets. The absence of a commercial market puts the entire financial burden of LLINs for both low- and middle-income households on the public sector and that burden can be overwhelming.

In 2016, governments and international partners spent US$ 2.7 billion on global malaria control and elimination (e.g. below $2 per person at risk of malaria) [4]. Out of the total, 74% was spent in Africa and the African governments’ contribution was 31%. These expenditure level would need to triple by 2030 in order to meet global malaria reduction targets [4, 7, 8]. Households also bear significant out-of-pocket (OOP) costs related to the treatment of malaria. For example, in 2014, Ghanaian households paid an average of $2.10 and $11.8 OOP in direct and indirect costs, respectively, per malaria treatment at formal health facilities [9]. Similarly, local businesses in high-burden countries are also affected by increased staff absenteeism and private healthcare costs.

Likewise, while the WHO predicted that 21 countries could eliminate malaria by 2020, 11 of these have shown marginal but consistent increases in malaria cases, and another 25 countries, mostly in Africa, show case increases of 20% [4, 6]. One contributory cause for stalled progress is a lack of sustainable and predictable funding [6]. As the stakes for controlling malaria increase, strengthening and identifying efficient strategies to widen funding sources for LLINs is crucial.

Little is known about the potential demand for high quality LLINs that could be sold in commercial markets, particularly among non-poor households. Three studies in Tanzania, Madagascar and India, using rigorous consumer preference measures, found low demand for LLINs among mostly poor-income households, but also indicated the potential for making demand stronger through micro-consumer loans and voucher subsidies [10–12]. The study in Tanzania also found strong demand (44%) for LLINs among a sub-group of least-poor households and significant willingness-to-pay for LLINs that matched consumer preferences for nets’ shape and size. However, the focus of the Tanzania study was not the non-poor households. Understanding what is the market potential among the non-poor households is important given that they make 52% of the population needing LLINs in high malaria risk countries in Africa and that they are the population more likely to create a sustainable commercial market of LLINs [13].

This study seeks to evaluate the demand and willingness-to-pay (WTP) for LLINs with characteristics that match consumer preferences among the middle-income households in a high malaria risk country such as Ghana. The study assesses whether there is a statistically significant demand for buying LLINs among middle-income Ghanaians and determines what LLIN features or “attributes” consumers find most attractive. The study also estimates how many LLINs are likely to sell and the government’s net costs and savings in a scenario where all LLINs are distributed free (i.e., only a public programme exists, “the status-quo”) *versus* a scenario in which only the poor get subsided LLINs (i.e., a public programme of free LLINs targeting poor households and a private market of improved LLINs targeting non-poor households coexists). Evidence from this study can inform decision-making whether private commercial markets can play a larger role in the continuous distribution of LLINs, help increase access to LLINs, and reduce funding gaps in malaria prevention.

## Methods

### Data

Estimates of the demand and WTP of LLINs were based on a representative sample of middle-income households from three regions in Ghana: Ashanti, Greater Accra, and Western. The evaluation used a discrete choice experiment (DCE) design with a real payment choice. A DCE is a quantitative technique based on conjoint-analysis theory that elicits consumer stated preferences for commodities from a sample population. The DCE technique was selected over other stated preference techniques, such as contingent valuation, because it allows for the valuation of trade-offs between multiple net characteristics or “attributes” (i.e., size, shape) and characteristic types or “attribute levels” (i.e., colour types: white, blue, green) [14]. DCEs are a widely applied approach in research associated with health commodities [15].

### Study population

The study targeted the regions in Ghana where people are at risk of contracting malaria, have the lowest saturation of household LLIN ownership and have purchasing power to buy their own LLINs. These three criteria ensured that the evaluation focused on the areas with the potential to capture a market share for a sustainable commercial market of LLIN. In 2017, the number of confirmed malaria cases in Ghana was 150 per 1000 people [16]. Out of the ten regions in Ghana, Ashanti, Greater Accra, and Western regions had high incidence of malaria ranging between 100 and more than 300 cases per 1000 people, with a very few small areas with lower rates) [16]. These three regions were also the most urbanized (64%, 92% and 45%, respectively) and had low LLIN ownership (70%, 61% and 67%, respectively, owning at least one LLIN) [17, 18]. The selection of areas with high purchasing power (i.e., the middle-income population) was based on areas with the lowest poverty rates. The poverty rates used to select regions and districts into the sampling strategy were those estimated by the Ghana Statistical Services (GSS) 2015 Ghana poverty mapping report [19]. The GSS estimates poverty at the district and lower levels of disaggregation based on estimates of per capita consumption by combining information from censuses and household consumption surveys. Ashanti, Greater Accra, and Western regions had the lowest poverty incidence (15%, 5.6%, and 21%, respectively) [17–19]. Households in these regions had a per capita income of at least US$4 (GHS 18) per day, with an average yearly household income of US$7775 (GHS 34,445), which is two times the yearly national average of household income, US$3757 (GHS 16,645) [17]. Within these three regions, the study focused on the 28 non-poor districts (i.e., districts with poverty rates lower than 9.6%) [19]. Individuals eligible to participate in the DCE were adult (18+ year olds) household members with knowledge about the use of bed nets and finances in the household.

### Sampling

A cross-sectional study design was used. Out of 1075 households recruited for a broader LLIN household survey [20] evaluating malaria ideation and LLIN usage among the same study population, a random sub-sample of 628 households were selected to take part in the DCE. The sampling frame used a stratified two-stage cluster sampling method, see Additional file 1: Appendix A for sampling details.

To evaluate household preferences between 13 different LLIN attribute levels and survey questions with 3 choice set alternatives in a DCE (see DCE sections below for details), the minimum sample size required was 600 respondents. This sample size provides sufficient statistical power for the DCE based on having a minimum of 50 respondents per alternative plus an additional 50 (3 × 50 + 50 = 200) [21, 22] and 200 participants per sub-group analysis (200 × 3 regions = 600) [23]. This sampling strategy has been used in prior DCE studies and meets other literature’s minimum sample size criterion of having at least 30 respondents for every level tested (13 levels × 30 = 390) [24, 25]. This statistical power is needed in order to statistically differentiate the effect of price between different attribute levels.

### Identifying the DCE attributes and assigning attribute levels

The selection of LLIN attributes and attribute levels included in the questionnaire was supported by data from a pilot DCE with 50 respondents and qualitative techniques collected from the same study population [26]. These mixed research methods were designed to understand what improvements to the LLIN attributes were the most desired and which were affordable and feasible for manufacturing. Qualitative techniques included focus groups (with 60 adults and 30 teenagers) employing both semi-structured opened-ended questions and human-centered design (HCD) [26]. These methods focused discussion to understand people’s facilitators and inhibitors to LLIN use and product preferences. Other qualitative methods included retail audit reviews, key informant interviews with LLIN supply chain importers and wholesalers, and recommendations from malaria programme experts [20, 27]. Evidence from the qualitative data was used to construct the list of LLIN attributes tested in the DCE, see Table 1 for the final list of the four attributes (i.e. shape, size, entry-design and price) and 13 levels. The attributes shape and entry-design each included 3 levels. The attribute size included two levels, and the price included 5 levels. The minimum and maximum prices tested were $1.10 (5 Ghanaian Cedi, GHS) and $14.30 (65 GHS), respectively. The maximum price was equivalent to a 25% margin above the estimated cost for manufacturing and distributing the LLIN with all the most expensive attribute levels (i.e. size queen, zipper entry, rectangular 4-point-hang). The maximum LLIN price estimate, $11.44 (GHS 52), was derived from information provided by retailers during retail audit interviews.

**Table 1:**
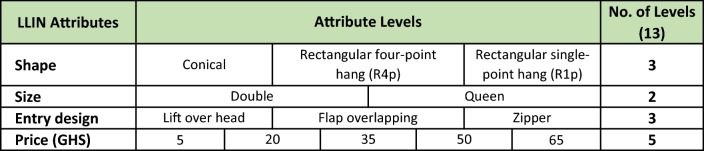
List of LLIN attributes and attribute levels

The 13 attribute levels were the final list out of 17 levels considered for the model. See Additional file 1 for details on the inclusion criteria for attribute levels

### Designing the DCE choice sets

The DCE survey instrument was composed of 18 choice questions. Each question provided participants three alternatives: whether to buy “LLIN A”, to buy “LLIN B”, or to buy neither “to opt out”. LLIN alternatives A and B each specified a level for each attribute tested in the model. The different types of LLINs that could be created combining 13 levels from four attributes is 90 (= 3 × 2 × 3 × 5). These 90 LLINs could be combined into (90 × (90 − 1)) = 8010 choice pairs (i.e. LLIN choice A or B pairs), known as the “Full Factorial Design”. This large array of choice questions was reduced to a manageable number using orthogonal fractional factorial design (FFD) [14, 28, 29]. FFD is a statistical technique commonly used for DCE designs that draws a small sample of choice-pairs such that each level appears enough times in the survey for the analysis to capture the effect of changes to each level on the probability of purchase (“LLIN demand”). The maximum and minimum number of survey questions recommended for a DCE, ensuring both the collection of enough data for drawing statistical inferences and the reduction of participant exhaustion, is between K/(J − 1) and 18, where K is the number of attributes (4) and J is the number of choice “alternatives” (A, B, Neither = 3), thus between 2 and 18 questions. As such, we designed a survey with the maximum of 18 choice questions to maximize statistical power, where 15 choice questions were used for the FFD and the remaining 3 choice questions were used to test for participants’ response rationality and consistency, see Additional file 1: Appendix B for details and the questionnaire design. The FFD was calculated using the statistical software R version 3.4.3.

Lastly, after the DCE, the survey also included questions about malaria ideation, reasons for bed net ownership and use as well as the use of insect sprays and the presence of air-conditioners in the home.

### Binding intention to buy the product

The DCE survey questions asked participants: “Which LLIN are you most likely to purchase: Bed Net A, Bed Net B, or Neither A nor B is preferred?” To mimic as close as possible an everyday purchasing situation, each participant was given money to elicit a validated “bidding” purchase choice (i.e., a true stated preference) instead of a hypothetical choice [27]. Each respondent received a cash payment of $14.30 (65GHS) in the local currency. This amount was sufficient to pay for the highest LLIN price in the survey. Thus, for each of the 18 choice questions, respondents knew they would be immediately able to buy any LLIN option if they wanted to buy it and retain any remaining change (the difference between $14.30 and the LLIN price specified in the alternative). Likewise, they were explicitly told they could opt out and keep all of the cash—just like in a real shopping situation.

It was not logistically possible for survey administrators to carry 18 types of nets with them in the field, thus only four real net types were stocked. The four net types in stock were the nets specified in two out of the 18 choice questions. At the end of the survey an electronically generated number randomized one out of the two survey questions with nets in stock. Survey administrators were blind to the randomly generated number. The respondent’s actual stated preference corresponding to the randomly chosen question was reviewed with them and based off their response the participant received either the Net A and the balance of remaining change, Net B and the balance of remaining change, or all of the cash payment and no net. Participants were alerted from the outset about how their stated preferences would be made binding and have real consequences.

Before the participants were administered the DCE survey, they were provided detailed contextual information about how the study procedures worked. LLIN attributes and levels were defined using both standardized text and 11 × 11 inch laminated cards with pictures, and the experiment was preceded by a practice mini-DCE using candies to help participants understand the exercise, see Additional file 1: Appendix B for details. During the experiment, each choice set was also illustrated in laminated cards with pictures making each choice set visual and facilitating comparison. DCE survey facilitators were trained in administrating the survey and answering participant questions. Participants signed consent forms and interviewers administered the DCE survey using electronic tablets. DCE survey questions appeared in random order and the order in which each question was answered for each participant was recorded and used in the analysis as a control for participant survey exhaustion. See Additional file 1: Appendix B for DCE design and procedure details. All human subject research activities were reviewed and approved by both the Johns Hopkins University and Ghana Health Service internal research review boards.

### Statistical strategy

Assessment of LLIN demand was done using multivariate logistic regression with random effects, a model of observation errors clustered by person-question-ID to correct for unobserved or random preference variation [23], with controls for attribute levels, competing alternatives, and respondents’ demographic and socio-economic characteristics, using the following equation:

$$\begin{aligned} BuysLLIN & = \beta_{0} + \beta_{1} Price + \beta_{2} Shape + \beta_{3} Size + \beta_{4} EntryDesign \\ & \quad + \beta_{5} OtherALTlevels + \beta_{6} SES + \beta_{7} HHmmbrs + \beta_{8} Qorder \\ & \quad + \beta_{9} InterviewID + \beta_{10} Price*Female + \beta_{11} Price*Rural + \varepsilon . \\ \end{aligned}$$

Although a respondent was making only 18 declarations of (A, B, or Neither), we can view the exercise as a set of 36 forced choice binary declarations of “Yes I would buy the option on this card” albeit each of these declarations was made in the context of a defined competing alternative. BuysLLIN is a binary response variable equal to one if the respondent’s choice was to buy the net and zero otherwise. Price is the LLIN price coded as a continuous variable. As shown in Table 1 above, shape is a vector of three levels, each coded as dummy variable, including rectangular 4-point hang (R4p), rectangular 1-point hang (R1p) and conical (omitted in the analysis as a base-level of comparison); size is a dummy equal to one for queen and zero for double; and entry design is a vector of three levels including lift-over-head, zipper, and the base-level flap-overlapping. Coefficients can be interpreted as the change in the probability of buying a LLIN with that attribute level compared to the base-level, holding other variables at their means.

Other ALT levels is a set of vectors of the Price, Shape, Size, and EntryDesign of the alternative card that was the context for the one that was under consideration. The model included various socio-economic and demographic variables about the respondent including: a sex dummy equal to one for females and zero otherwise, a secondary education dummy, a married dummy, a vector of three region variables, including Ashanti, Western and the base-level Greater Accra, a SES vector of five wealth dummies (all pertaining to “non-poor” Ghanaians) where the base-level is the lowest wealth, and a dummy for residency type equal to one if rural. HHmmbrs is a continuous variable on the number of household members. Qorder is a continuous variable on the order in which that survey question appeared, InterviwerID is a vector of all 12 survey interviewer ID dummies added to control for the influence of individual interviewers on the choice to buy, and lastly, Female*Price and Rural*Price are interaction terms on the difference between the price females and males pay, and between the price individuals in rural and urban areas pay, respectively.

The error term, ε, was modelled as a random intercept that could be decomposed into components from within the individual and within a particular card set to account for the non-independence of observations that were clustered.

Three of the 18 questions were planted only to test for invalid responses (e.g., they contained “no brainer” options where one alternative was superior across all domains). The analysis was run on the subset of 15 questions from the FFD. The logistic regression coefficient values were converted to marginal effects to ease their interpretation as elasticities of the probability of purchase. Demand curves plotted the predicted probability of purchase from the model vs. price. Estimates of average total revenue (ATR) at any given price was calculated as the product of price times probability of purchase for every 100 individuals encountering an opportunity to purchase. Over 50 regression specifications were tested adding one SES variable a time and testing consistency of results. The robustness of results were also explored removing the irrational, inconsistent or always-buyers (“disengaged”) responses [23]. Probability of purchase and WTP estimates were stratified by key LLIN attributes and individual characteristics. Similarly, the probability of purchase for both the least and most attractive LLINs were estimated at the average WTP price as well as at the low and high price points of $4.40 and $13.20 (GHS 20 and 60). The analysis was computed using STATA software version 14.

Lastly, we used a microsimulation model to estimate the total public cost and coverage outcomes under two policy scenarios. In scenario one, the public sector buys at least one LLIN for households in a defined population. In scenario two, there is a commercial market that conforms to the WTP estimated by the model. A Monte Carlo simulation with 1,000,000 iterations of the model was run to produce confidence intervals (CI) around cost and savings estimates.

## Results

### Descriptive statistics

Among the 628 DCE participants, a majority were females (61.3%), had secondary education (70.0%), and were heads of the household (75.2%), see Table 2. About half of the population were over age 35 and married (52.2%), most lived in urban areas (89.2%) and were employed. The average household size was 3.1 members and the majority of households (69.3%) did not have a bed net. The average net ownership was 1.7 nets per household. Among the minority (30.7%) with at least one bed net only 0.9% of them reported using it. The majority of the study population believed that malaria is not easy to treat, that insecticide-treated nets are effective and that it is easier to get a good night sleep when sleeping under a bed net. But, they also believed that it is difficult to sleep well under a bed net when the weather is warm. See Additional file 1: Appendix C for more results on net ownership and malaria ideation.

**Table 2:**
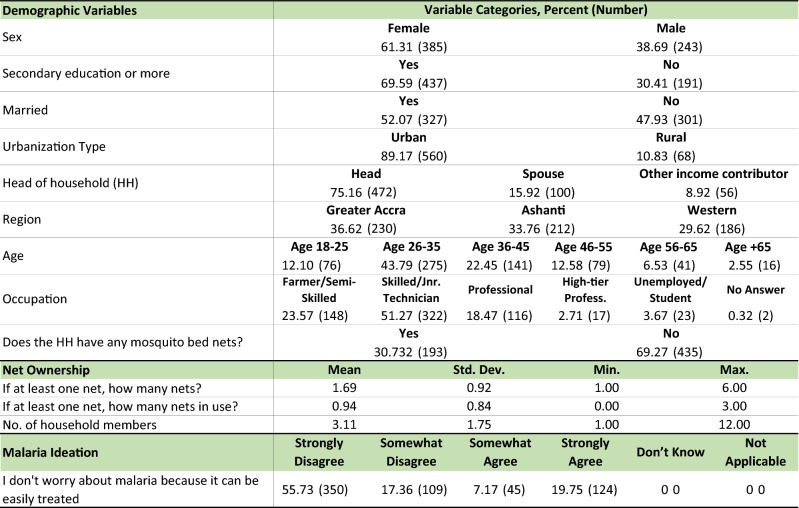
Study population descriptive statistics, total n = 628

Participants were also asked about the reasons for not owning a bed net and ownership of other mosquito control products (results not listed in the table). The main reasons for not owning a bed net were use of other malaria control products (58%), the weather was too hot for using them (32%), and not getting a LLIN during the mass campaign (17%). Less than 10% of the households mentioned other reasons for not owning a bed net, including could not afford it, it feels restrictive, has adverse health reactions and gets worn out. A total of 82% of the study participants used other mosquito control products including air conditioning, electric fans, aerosol insecticide sprays and coils. Out of the study population, about one-fifth (19%) had air conditioners in their household, 5% of them used the air conditioners exclusively to protect against mosquito bites and none of the households used LLINs. Out of the study population, 94% had electric fans in the household, one-fifth (18%) of them used the electric fans exclusively to protect against mosquito bites, and a very small percentage (0.2%) used both LLINs and electric fans complementarily. Likewise, 50% of the air-conditioner users and 61% of the electric fan users used these mosquito control products instead of LLINs because these were easier to use. Another 50% of the air-conditioner users and 34% of the electric fan users used these mosquito control products instead of LLINs because they believed those were more effective preventing mosquito bites. Aerosol insecticide sprays were the most widely non-LLIN malaria control product used (61%) among the study population. Out of the households using insecticide sprays, 24%, 56% and 20% used them because they believed that compared to LLINs those were more effective controlling against Malaria, easier to use and more affordable, respectively. The majority of mosquito coils and repellent users believed that those products where either easier to use or more affordable than LLINs.

### Internal validity

Internal validity tests revealed that 9.24% (58), 5.41% (34) and 48.25% (303) of participants made choices that were irrational, inconsistent or anchored to buying every choice (“always-buyers”), respectively. See Additional file 1: Appendix C Tables S2 and S3 for details. Knowing that some DCE respondents violated assumptions about rationality and consistency led us to restricted results the sub-sample of 541 respondents who were neither irrational nor inconsistent.

### The LLIN demand curve

The sample had an overall probability of purchasing a LLIN of 43.8%. On average, purchasers were willing to pay $7.48 (GHS 34.0) for a LLIN across all presented attributes. Based on these results, for every 100 middle-income Ghanaian households in the study areas, the average total revenue (ATR = price x quantity) from LLINs purchased would be $327.49 (or GHS 1488.61). The difference in the mean probability of purchase between those who already owned a LLIN and those who did not own at least one LLIN was a 1.5% percentage point difference (*p* value < 0.00). As expected, regression results showed that price negatively affected the probability of LLIN purchase, see Table 3. For every price increase of $0.22 (or GHS 1), the probability of purchase decreased by an average of 0.13% (p < 0.00), holding all other net attribute levels and respondent characteristics at their means.

**Table 3:**
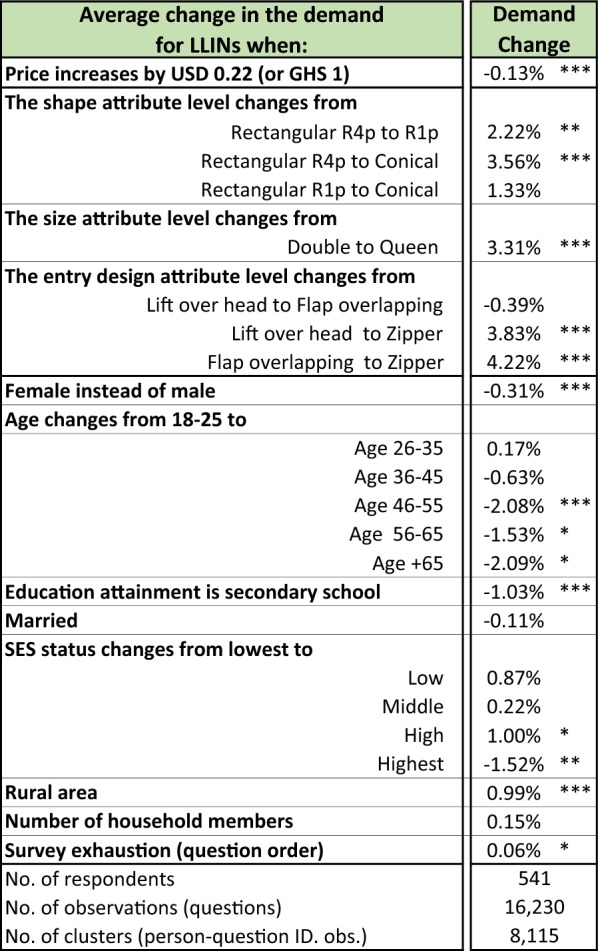
Estimate of the DCE demand model

R4p and R1p is rectangular 4-point hang and 1-point hang, respectively. The number of asterisks indicates the level of statistical significance where: *** is p-value < 0.00, ** is p-value < 0.05, * is p-value 0.10, and no asterisk means not statistically significant. The list of interviewer dummies is not shown to reduce the size of the table. The marginal effect for each region and price interactions were not estimable. However, we show results for each region in the table of stratified analysis

**Fig. 1:**
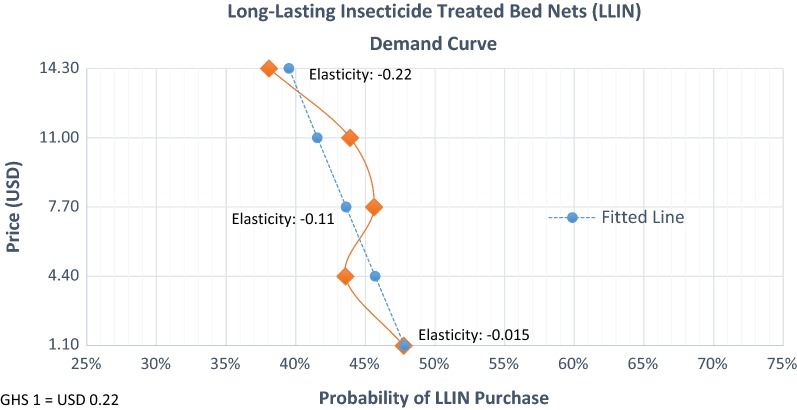
Long-lasting insecticide-treated bed nets (LLIN) demand curve. Note: The dotted line is the mean linearly fitted demand probabilities. The solid line are the raw data (responses from the 628 respondents to each of 15 choice questions)

Figure 1 shows that within the range of prices tested in the DCE, $1.10–14.30 (GHS 5.0–65.0), the price elasticity of demand was inelastic, with an average elasticity of − 0.11 (C.I. − 0.087 to − 0.13). This means that changes in the price only produce modest changes in the quantity demanded. An elasticity of − 0.11 means that a one percent increase in the LLINs’ price would decrease demand by 0.11% (on average, a one percent price hike is $0.07 or GHS 0.34). Because of the inelasticity, in a hypothetical population of 100 middle-income households, increasing the price by one percentage points above the average would increase total revenue from $327.49 to $366.85. Likewise, the proportion of respondents willing to pay the highest price tested in the analysis, $14.30 (GHS 65.0), was only slightly lower than the proportion willing to pay for the average WTP (39.5% vs. 43.8%). At the highest price tested, $14.30, the average total revenue is $565.06 (or GHS 2,568,44) per 100 households. See Additional file 1: Appendix Tables S4a, b for tabulations of demand probabilities and price elasticities of demand by WTP.

### Effect of attribute changes on demand

Table 3 also shows the effect of substituting levels on the probability of purchase. The tradeoff between shape R4p and R1p, or from R4p to conical, increased the average probability of purchase by 2.22% (p < 0.05) and 3.56% (p < 0.00) percentage points, respectively. The difference in the probability of purchase between R1p and conical was not statistically significantly different. Increasing net size from double to queen increased demand by 3.31% (p < 0.00) and changing the net entry design from either lift-over-head to zipper, or from flap-overlapping to zipper, increased demand by 3.83% (p < 0.00) and 4.22% (p < 0.00), respectively. The difference in the demand between lift-over-head and flap-overlapping was not statistically significantly different.

### Effect of respondent characteristics on demand

Various sociodemographic characteristics also changed demand for LLINs. Males’ had a slight 0.31% percentage point (p < 0.00) higher demand than females. Demand by those 18–25 years old was also slightly higher, by 2.08% percentage points (p < 0.10 and 0.00), compared to those 46 and older. Having secondary education or being from the highest income group (within our middle-income population sample) slightly decreased demand by 1.03% (p < 0.00) and 1.52% (p ≪ 0.05), respectively. Living in a rural area increased demand by 0.99 percentage points (p < 0.00).

### Sensitivity analysis of demand changes

Some analysts believe that real consumers exhibit features of non-rationality and anchoring in their market behavior. Thus, additional models were examined to assess whether the results would change by including responses from the respondents who showed irrational or inconsistent choice behaviour. In general, findings were very similar to the main results, see Additional file 1: Appendix C Table S5.

Similarly, models excluding the sub-group (n = 303) who anchored to always buying in all 18 choice-sets were also examined. Excluding the always-buyers made the shape level R1p not statistically preferable to R4p (i.e., only conical would be the preferred shape) and all other results remained constant, see Additional file 1: Appendix C for details. In our main results, including the always-buyers, both the R1p and conical shapes were preferred to the R4p. This may indicate that the R1p feature was especially appealing to this subset of always-buyers.

### Stratification of analysis by sub-populations

Sub-group analysis of individuals with and without LLIN in the household, females *vs.* males, and urban vs. non-urban were generally the same with demand elasticities ranging between 0.04 and 0.15% (p-values < 0.00). See Additional file 1: Appendix C Table S6.

### Attribute combinations most and least likely to increase LLIN demand

**Fig. 2:**
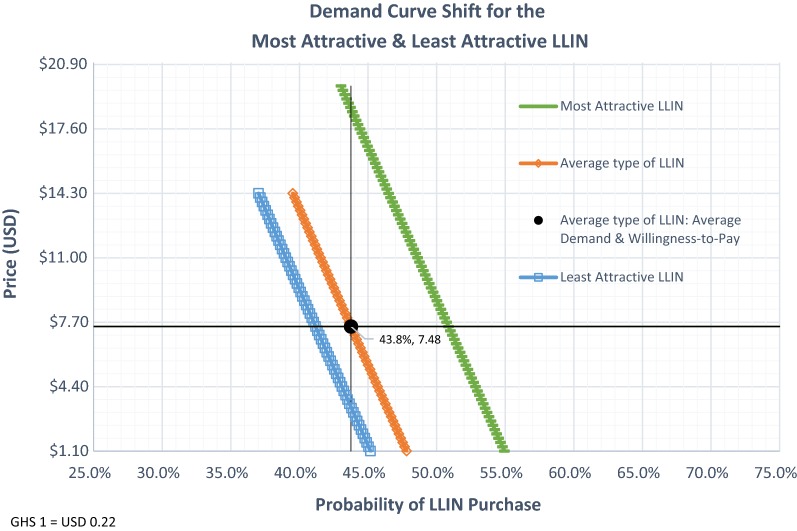
Demand shifts. Notes: Lines are mean linearly fitted demand probabilities using multivariate regressions and margins

Changing LLIN attributes from the least attractive levels (e.g., R4p, double size, lift or flap entry) to the most attractive (e.g., conical or R1p, queen size, and a zipper entry) shifts the demand curve to the right, see Fig. 2. With this shift, holding the average probability of purchase constant (43.8%), the average WTP increased from $3.30 (GHS 15) to $18.48 (GHS 84), respectively. Re-engineering product attributes from the least attractive, which are the most common type of nets given through free distribution channels, to the most attractive LLIN would improve ATR from $144.48 (GHS 656.74) to $809.10 (GHS 3677.73) per every 100 households.

### Public cost savings and commercial market revenue

Given that the data sample is representative of all the 2.6 million households living in the three study regions (e.g. 32 districts with lower than the average poverty rate: 0.7–9.6%) it is possible to extrapolate data from the sample to the population in these regions. Assuming that current LLIN coverage levels are met and the cost per LLIN is the manufacturers’ price of $4.38 [20], the total cost of providing one standard unenhanced LLIN to each current owner (0.87 million households) would be $3.7 million in 2017 USD, see Additional file 1: Appendix Figure S1a and Table S7a, b for model calculations and parameters. In an alternative scenario in which the commercial market offers an enhanced LLIN for sale at a price of $7.48 per LLIN, we project private sales of LLINs producing revenue valued at $8.1 million (95% C.I. 5.9 –10.6 M) from 1.08 million non-poor households (or $311 per every 100 households in the study area). Revenue from private sales would support jobs for Ghanaian retailers, distributors, and manufacturers of LLINs. Total LLIN coverage would increase by 85% from new LLIN owners from non-poor households who did not already have a LLIN. Of note, we project that the public sector outlay would be reduced by 39% (95% C.I. 50.30–27.89%), from $3.7 million to $2.3 million, from people who are now buying their own LLINs, rather than relying on the ones given through free distribution. Estimates assume a conservative scenario in which poor households do not buy LLINs. The Additional file 1: Appendix Figure S1c shows additional estimates for scenarios in which LLINs are purchased to close the LLIN coverage gap as opposed to meeting current coverage levels.

## Discussion

The results support a scenario in which commercial markets for LLINs could play a significant role in improving access to LLINs for non-poor Ghanaians in malaria-endemic areas. Despite low bed net ownership and significant ownership of non-LLIN malaria control products the discrete choice experiment showed strong demand and high willingness to pay for improved LLINs. Low ownership and use of LLINs among the population may be due to low supply of nets that consumers like [20]. For instance, only 7% [20] of local markets in the study area sold nets and those most commonly available had the combination of characteristics that were the least preferred out of all the attributes tested in the DCE (i.e., rectangular 4-point-hang, size double, and lift overhead entry LLINs).

The study showed that the average demand for LLINs with a variety of attributes is strong among both current net owners (44.8%) and non-net owners (43.3%) asserting and demonstrating a high willingness to purchase. The study validated their statements by observing not just their statements in a DCE but their actual purchases with money that they could have kept for any alternative use. Likewise, participants’ mean willingness-to-pay, $7.48, was much higher than the mean price of $4.38 for imported nets currently sold in local markets [20]. The price elasticity for LLINs was inelastic, thus, changes in price around the range tested will not significantly decrease demand. However, the price elasticity of demand may be elastic (see limitations below) at prices higher than the price tested in this analysis (e.g., $14.30). Marketing LLINs with the most attractive attributes has the potential to increase average demand and WTP by up to 8.27% percentage points and $18.48, respectively. Results also indicate that individual characteristics, such as living in a rural area, being male, being less wealthy and living in the Ashanti Region were also associated with higher LLIN demand.

The private and public commercial markets for LLINs would augment each other offering different products and targeting different segments of the population. The private market would develop if suppliers focus on addressing the demands of the middle-income population. The public market of free LLINs could then continue meeting the public sector’s demands for LLINs distributed to low-income populations with freed up resources. Together, the private and public strategies encouraging commercial markets for LLINs will help increase the population’s access to LLINs as long as there remains a public commitment to ensure access to free LLINs for those for who cannot pay for them out-of-pocket. Likewise, building a stronger private commercial market for the LLINs with improved attributes could spare the public sector more than half of the cost of supplying the current free LLINs to individuals who have the means and willingness to buy LLINs with improved features. The public sector could increase the low-income populations’ access to LLINs by allocating newly freed up funds to either expand free LLINs distribution campaigns or subsidize LLINs available in the private market. The demand exists to generate substantial revenue for a private commercial market. This market would also benefit society by creating new economic opportunities for local Ghanaians retailers. The strong demand for LLINs also has the potential to increase LLIN usage because middle-income households would be acquiring a product that they like and thus be more likely to use it.

### Other studies

Six studies in sub-Saharan Africa have looked at the WTP of LLINs, including one from Ghana. All studies find a negative association between price and demand, but some find different levels of demand and WTP. Some of these studies used less robust preference-based study designs that suffer from significant bias for estimating WTP, targeted poor households, or tested different products, making results incomparable. For example, Gingrich et al. using a DCE in Tanzania (testing preferences for insecticide-treatment, shape, and size) also found significant demand for LLINs, inelastic price elasticity (among both poor and least poor populations), increased demand for specialty features (e.g. rectangular, larger size, and insecticide-treatment), and higher demand in rural areas [12]. However, their study found a much lower WTP (between $0.5 and 1.4). The difference between the studies’ results may be explained by differences in the study designs. Gingrich et al. capped prices tested much lower (8000TSH or about $3.5 vs. $14.30 in our study) and focused on poor-income groups–which have lowered WTP, and this study tested other new improved net features.

Another study in Ghana using an auction study design found significant WTP for a solar-panel net-fan (about $13), suggesting that their fan product could be a complementary good for bed nets, presumably also increasing demand for bed nets [30]. However, they did not measure WTP for bed nets.

Two other studies, in Ethiopia [31] and Nigeria [32], found large demand for LLIN for low WTP values and an elastic WTP. However, both studies employed direct consumer survey stated preference techniques which are less robust than conjoint analysis [33]. Another study in Madagascar by Comfort and Krezanoski, using revealed preference data from a RCT field experiment and subsidizing LLINs at 0%, 25%, 50%, 75% and 100% for a maximum cost of $2.20 among low-income groups, found a very elastic price of demand for higher prices (i.e. for each $0.55 increment, demand decreased by 23.1% points) [10]. Similarly, Tarozzi et al. using a field experiment in India among poor individuals found very elastic prices of demand for LLINs (i.e., demand decreased by 50% when the price increased by 20%) [11]. Likewise, Dupas using subsidies in a field experiment in Kenya found elastic LLIN prices [34]. These three field experiments are consistent with prior literature by Cohen and Dupas indicating high price elasticity of demand for health products among poor households [35]. The diminished price sensitivity of the study’s sample is to be expected precisely because the population intentionally sampled were the non-poor households for whom the next best use of funds was much less likely to be an essential need.

### Limitations

The results are unlike the prior studies that included poor households, for which the price elasticity of demand for LLINs was typically elastic. These results are consistent with higher-income individuals being less responsive to changes in prices than low-income individuals [36]. However, in this study, estimates are restricted by the top LLIN price tested, $14.30. It is possible that higher prices than those tested would have revealed more sensitivity to the price. Future experiments involving middle-income populations would need to incorporate wider price ranges to determine the price elasticity at higher LLIN prices [37].

DCEs can be limited when there is poor respondent understanding of attribute levels or cognitive fatigue. However, this bias was mitigated by piloting the DCE so that the study instruments could improve contextual information. The study also used visual aids, illustrating each of the 18 question choices and each attribute level. The DCE module occurred early in the household survey modules to reduce respondent fatigue and included a short 2-question practice DCE before administrating the actual DCE to respondents. Similarly, survey administrators reported in general having no issues with participants’ understanding of the various attribute levels or the exercise. Likewise, to mitigate the influence of priming respondents about the importance of malaria, which could bias results upward, we administered the malaria ideation question after the DCE.

Likewise, prior studies have suggested that providing a cash transfer might bias choice behaviour upward toward buying. However, the study design took pains to emphasize that the respondent could absolutely keep the endowment if they did not spend it on the purchase of a net. The presence of the cash transfer in the study was a necessary component for this DCE to validate stated preferences with observed real market behaviour [11, 33, 38].

Also, a sub-set of the study population behaved unexpectedly in that they anchored to buying a net for all 18 choice-questions in the experiment. Such behaviour could bias choice data if participants are not providing their actual preference between the various net features. Excluding the 303 households anchoring to always buying made the shape rectangular-1-point-hang not statistically preferable indicating that this feature is especially appealing to this subset of always-buyers. Other than this difference, sensitivity analysis showed results where robust to the removal of either irrational respondents or always-buyers.

Lastly, out of all the attributes tested in the DCE, the rectangular-1-point-hang was the only attribute that participants did not have experience with prior to the DCE, given that the idea for a rectangular-1-point-hang came directly from the qualitative work preceding the DCE. This unfamiliarity with the design may have also impacted the results.

An important strength of this study is that the respondents knew there was money at stake. They knew they would have to back up their statements of willingness to purchase LLINs with actual purchases of the nets they said they would purchase at the prices they agreed to. They knew they could leave the encounter with cash payments if they felt they were better off with having cash instead of immediately using the cash to purchase an LLIN. This design feature improves the internal validity and helps us support claims that stated preferences and willingness to purchase are reflective of actual revealed preferences about how people would spend their own money on. However, there may still be some leftover social acceptability bias that may have skewed some respondents to be more willing to spend their new cash on the offer of a LLIN.

## Conclusion

Using rigorous techniques from a sample of middle-income households in Ghana, this study shows that there is a strong demand for LLINs in the private market, particularly for LLINs with the characteristics that meet consumers’ preferences. This evidence shows that the private commercial sector could be a viable channel for distributing LLINs. A private market would augment the public market. It could help increase LLIN coverage, and help the public sector save money from households buying their own nets. Evidence from this study may help manufacturers and retailers better understand the revenue opportunities linked to supplying key consumer-preferred LLINs. The public and donor sectors should incorporate policies into their national malaria prevention plans supporting commercial markets for consumer-preferred LLINs. However, policies would have to remain in place that assists poor households in acquiring LLINs so that an equitable market could combine both efficiency and fairness.

## Supplementary information

**Additional file 1.** The appendix supporting the conclusions of this article is included within the article (“LLIN DCE Appendix”).

## Data Availability

The data are not publicly available due to institutional review board restrictions because the data contain information that could compromise research participant privacy/consent.

## Abbreviations

LLIN: long-lasting insecticidal bed nets
DALY: disability-adjusted-life-year
ITN: insecticide-treated net
GTS: Global Technical Strategy
WHO: World Health Organization
WTP: willingness-to-pay
DCE: discrete choice experiment
GSS: Ghana Statistical Services
R4p: rectangular four-point hang
R1p: rectangular single-point hang
HCD: human-centered design
GHS: Ghanaian Cedi
FFD: fractional factorial design
SES: socio-demographic variables
ATR: total revenue
CI: confidence interval
