# Supplementary material for: Willingness-to-pay for long-lasting insecticide-treated bed nets: a discrete choice experiment with real payment in Ghana
Source: Malar J. 2020 Jan 13;19:14. doi: 10.1186/s12936-019-3082-6 (PMC6958784; doi:10.1186/s12936-019-3082-6)
Supplement: Supplementary file 1 — Additional file 1. The appendix supporting the conclusions of this article is included within the article (“LLIN DCE Appendix”). [file 12936_2019_3082_MOESM1_ESM.docx]

APPENDIX

Willingness to pay for long-lasting insecticide treated bed nets: a discrete choice experiment with real payment in Ghana

**Contents**

[A. HOUSEHOLD SURVEY SAMPLING 2](#_Toc27343398)

[B. DCE DESIGN 2](#_Toc27343399)

[Criteria for selecting net attributes and attribute levels 2](#_Toc27343400)

[Picture of new LLIN design 2](#_Toc27343401)

[Table 1. DCE Survey Questions and Fractional Factorial Design 3](#_Toc27343402)

[DCE questionnaire rational and consistent tests 4](#_Toc27343403)

[Binding intention to buy the product 4](#_Toc27343404)

[DCE procedures 4](#_Toc27343405)

[C. RESULTS 4](#_Toc27343406)

[Household vs. DCE descriptive statistics 4](#_Toc27343407)

[Net ownership and malaria ideation 5](#_Toc27343408)

[What and who are the internal valid respondents 5](#_Toc27343409)

[Table 2. Internal validity tests: participants' rational and consistent question tests 5](#_Toc27343410)

[Table 3. Always-Buyers 5](#_Toc27343411)

[LLIN Purchase Probabilities by Price for Different LLIN types 6](#_Toc27343412)

[Table 4a. LLIN Average Probability of Demand by Price & LLIN Type 8](#_Toc27343413)

[Table 4b. LLIN Price Elasticity of Demand by Price Point 9](#_Toc27343414)

[Invalid Respondents & Sensitivity Analysis of Regression Analysis 9](#_Toc27343415)

[Table 5b. Sensitivity Analysis of Regression analysis 10](#_Toc27343416)

[Regression Analysis Stratification 11](#_Toc27343417)

[Table 6. Regression analysis stratified by sup-populations 12](#_Toc27343418)

[Public cost savings and commercial market revenue 13](#_Toc27343419)

[Table 7a. Model Parameters 14](#_Toc27343420)

[Table 7b. Poverty estimates by district 15](#_Toc27343421)

[Figure 1a. Policy Analysis Model – Estimates for all 2.6 Million Households in the Study Areas (Estimates Cost to Meet Current Coverage Levels) 16](#_Toc27343422)

[Table 7c. Summary of Key Results, Policy Analysis Model – Estimates for all 2.6 Million Households in the Study Areas (Estimates Cost to Meet Current Coverage Levels) 17](#_Toc27343423)

[Figure 1b. Policy Analysis Model – Estimates for all 2.6 Million Households in the Study Areas (Estimates Additional Cost to Close the Current Coverage Level Gap) 17](#_Toc27343424)

[Figure 1c. Policy Analysis Model – Estimates for a Random Sample of 100 Households in the Study Areas 18](#_Toc27343425)

[References 19](#_Toc27343426)

# HOUSEHOLD SURVEY SAMPLING

The sampling strategy implemented a stratified two stage cluster sampling method and is the same used for the 2016 Ghana Malaria Indicator Survey (GMIS) as well as the 2010 Population and Housing Census (PHC) conducted by the Ghana Statistical Service (GSS) of the Government of Ghana^1^. The only difference between the GSS and this study’s methodology was that, based on this study’s criterion, the sampling frame only included the three regions of interest and the 28 non-poor districts allowing for a total sampling pool of 1,511,369 HHs in the year 2017 out of 2,943,183 HHs in the three regions. The frame was a complete list of all census enumeration areas (EAs) created for the 2010 Population and Housing Census. The average size of the urban and rural EAs was 185 and 144 HHs, respectively. All EAs within the 28 non-poor districts were separated into residence types (urban or rural) and into the three regions; this yielded 6 sampling strata. Then, EAs were selected independently in each stratum achieving implicit stratification with the probability of selection proportional to the size of the EAs. Within each village, 15 HHs were systematically randomly selected. Power calculations required a total of 1,060 HHs to obtain estimates of the proportion of people using Insecticide Treated Nets (ITN) in each stratum with a 95% confidence interval (CI). This sampling frame yielded 71 EAs. These were 59 Urban and 12 Rural. The EAs were 26 from Ashanti, 20 from Greater Accra, and 25 from Western region. The 71 EAs had 1,060 HHs (15 HHs per EA). An oversampling of 140 HHs gave an allowance for HHs that were not available to be missed without requiring replacement and this ensured that the minimum sample requirement was met. The survey interviewers were asked to interview only the pre-selected households and no further replacements or changes were allowed in order to prevent sampling bias. The survey was administered using tablets and an electronic web based survey. The tablets’ electronic system randomized the household survey participants to either include or exclude the discrete choice experiment study component.

# DCE DESIGN

## Criteria for selecting net attributes and attribute levels

An original list of 17 attributes (e.g. shape) levels (e.g. small, medium, etc.) was considered for evaluation in the DCE. The original list included the 13 listed in the manuscript table 1. Originally there were also 3 levels for the installation technique attributes: nail hook, do-it-yourself, and a renter friendly hook design with no-screws-no-bolts levels. There was also an additional size attribute which was the level twin. Data from the DCE pilot and qualitative techniques narrowed down the levels from 17 to 13. The criteria for selecting attribute levels into the study included, either: importance for the 18 and older Ghanaian population, manufacturing ease, no overlap between levels (or clear participant understanding of differences based on pilot results), and statistical significance in the pilot study. Certainty of manufacturing ease was verified with global manufacturers during key informant interviews. The selection of the maximum price level (GHS 65) was based on a 25% price margin above the total cost for manufacturing, distributing and storing the net with the most expensive attribute combination. The lowest price level (GHS 5) was selected based on the lowest price available in the market which belonged to a second-hand bed net. Also, all participants were informed that all nets included in the DCE study were long-lasting-insecticide-treated bed nets, polyester material and white color.

## Picture of new LLIN design

The picture below illustrates a LLIN with two attributes: the zipper entry design and the rectangular one-point hang shape. Out of all the 13 attributes tested in the DCE, these two attributes were not generally found in the market. The picture helps visualize what a LLIN with these 2 novel attributes look like.


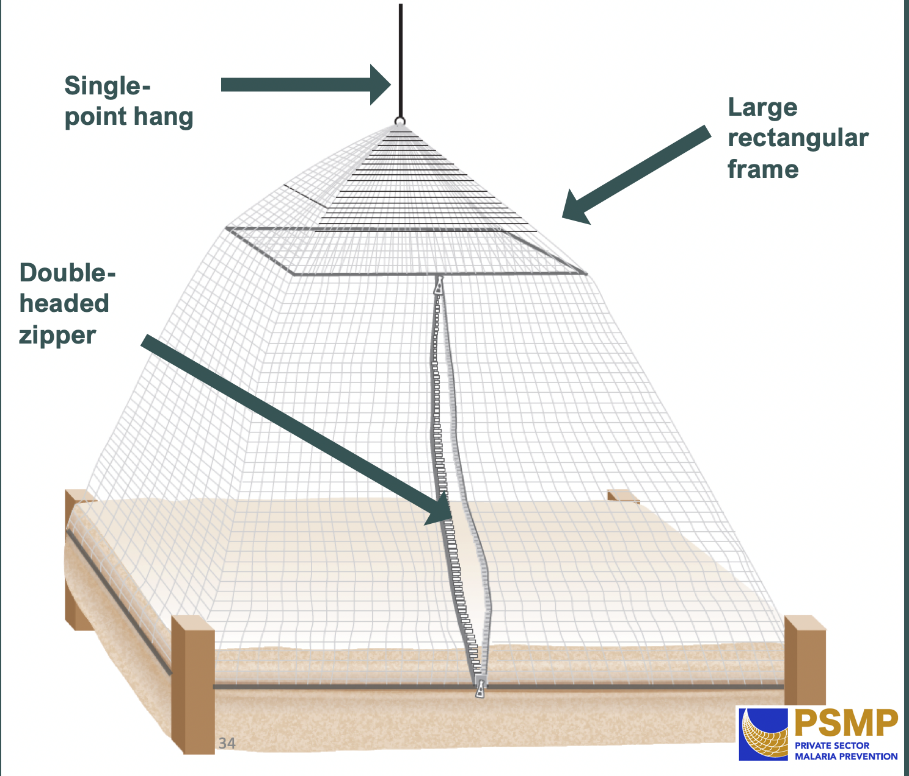


Table 1. DCE Survey Questions and Fractional Factorial Design

| Survey Question  No. ID |  | Bed Net Choice A | | | |  | Buy Bed Net Choice B | | | |
| --- | --- | --- | --- | --- | --- | --- | --- | --- | --- | --- |
|  |  | Shape | Size | Entry | Price |  | Shape | Size | Entry | Price |
| 1 |  | 1pRectangular | Double | Flap | 20 |  | 1pRectangular | Queen | Flap | 5 |
| 2 |  | Conical1p | Queen | Lift | 20 |  | Conical1p | Double | Zipper | 5 |
| 3 |  | 4pRectangular | Double | Lift | 5 |  | 1pRectangular | Double | Zipper | 35 |
| 4 |  | 1pRectangular | Queen | Lift | 50 |  | Conical1p | Queen | Zipper | 65 |
| 5 |  | Conical1p | Queen | Zipper | 65 |  | 4pRectangular | Double | Flap | 65 |
| 6 |  | Conical1p | Double | Lift | 35 |  | 1pRectangular | Double | Lift | 65 |
| 7 |  | 4pRectangular | Queen | Zipper | 50 |  | Conical1p | Double | Flap | 50 |
| 8 |  | Conical1p | Double | Zipper | 5 |  | 4pRectangular | Double | Lift | 5 |
| 9 |  | Conical1p | Double | Flap | 50 |  | 4pRectangular | Queen | Zipper | 50 |
| 10 |  | 1pRectangular | Queen | Flap | 5 |  | Conical1p | Double | Lift | 35 |
| 11 |  | 1pRectangular | Double | Zipper | 35 |  | 4pRectangular | Double | Zipper | 20 |
| 12 |  | 1pRectangular | Double | Lift | 65 |  | 4pRectangular | Queen | Flap | 35 |
| 13 |  | 4pRectangular | Double | Flap | 65 |  | 1pRectangular | Queen | Lift | 50 |
| 14 |  | 4pRectangular | Queen | Flap | 35 |  | Conical1p | Queen | Lift | 20 |
| 15 |  | 4pRectangular | Double | Zipper | 20 |  | 1pRectangular | Double | Flap | 20 |
|  |  |  |  |  |  |  |  |  |  |  |
| 16 |  | 4p-Rectangular | Double | Zipper | 35 |  | 4p-Rectangular | Double | Zipper | 50 |
| 17 |  | 4p-Rectangular | Queen | Lift | 35 |  | 4p-Rectangular | Double | Lift | 20 |
| 18 |  | Conical1p | Double | Lift | 20 |  | Conical1p | Queen | Lift | 20 |

Note: 1p and 14 refers to the 1-point or 2-point rectangular shapes, respectively.

## DCE questionnaire rational and consistent tests

Three questions that tested for rational and consistent respondent answers were added to the 15-question FFD for a total of 18 DCE survey questions. Question 16 was a rational respondent test and questions 17 and 18 were consistency tests.

## Binding intention to buy the product

Research shows that participants may state their choice preference differently depending on whether the intention to buy the product is binding or hypothetical. Hypothetical willingness to pay is typically biased upward relative to binding responses. To ensure that the questions more likely elicited a realistic “bidding” response (i.e. a true preference) instead of a hypothetical choice, each participant was given 65GHS to cover the cost of net choice and then they were given the net of their choice at the end of the visit. If their choice was Neither A nor B nets, the participant kept all the money, and if their choice was a net, they paid the price of the net to the survey interviewer, received the net of their choice, and kept the change (the difference between the net price and the GHS 65).

## DCE procedures

To help the participants understand the DCE exercise prior to answering the DCE survey questions, the participants were first presented with a mini DCE about choice preferences between candies. This mini practice DCE asked two survey questions about candy preferences. For the candy exercise, each participant was given GHS 5 and asked “Which candy are you most likely to purchase: Candy A, Candy B, or Neither Candy A nor Candy B is preferred?”. Each candy alternative included a distinct price and flavor attribute. Then, they were presented with their choice, either the Candy of choice and change, or the total GHS5 if the choice was neither. This practice exercise helped to demonstrate that the stated preferences would be followed by a consequential resource transfer.

Likewise, to facilitate comprehension of each of the product attributes and the differences between the attribute levels, for each DCE survey question, the interviewer repeated the question and read each of the distinct attribute levels for each alternative while also pointing to the corresponding level picture illustrated in a standardized 8x11 laminated card. Thus, each interviewer had 18 3-card sets, each set of three cards illustrated the attributes one-by-one for net A, the same for net B and the last card was blank and read “neither bet net A nor bed net B” in big bold letters. The pictures were designed, edited and approved by five LLIN program managers and revised based on comments from the 50 pilot participants.

The average time for the DCE section of the survey (excluding the non-DCE sections of the household survey) was one hour and thirteen minutes. To reduce respondent survey exhaustion and biased results due to disengaged respondents, the DCE section was placed among the first modules of the HHs. Likewise, to control for potential bias in participant’s responses due to survey exhaustion, the survey questions appearing-order changed randomly for each participant and the appearing-order was added as a control parameter in the statistical analyses.

# RESULTS

## Household vs. DCE descriptive statistics

This main text reports on the sub-set of DCE respondents. However, data from both the HH and DCE are very similar. A separate report available from the authors can provide details^[[1]](#footnote-1)^.

## Net ownership and malaria ideation

A full list of net ownership and malaria ideation questions and answers can be provided upon request in the Market Research Report^[[2]](#footnote-2)^.

## What and who are the internal valid respondents

*Irrational Respondents:*

Scenario 16 was included to test rationality: both net choices included identical attribute levels expect net B was costlier than net A. Internal validity tests of the DCE survey responses revealed that out of the 535 respondents who chose to buy a LLIN for scenario 16 there were 476 (89%) who made a rational choice by choosing to buy “net A” which was the less costly net. The remaining 11% (or 59) were irrational, see Appendix Table 2.

*Inconsistent Respondents:*

Scenario 17 and 18 tested consistency because question 17 had an option that required a higher price for a larger net and question 18 offered a choice where everything was the same except that one choice had a larger net. Consistency required that those who paid more for a larger net would also prefer the larger net in question 18. Table 2 shows that out of the 596 who chose to buy a net for scenario 17, 49% (or 292) chose Net A (they chose to pay more for a larger net). Out of these 292 individuals willing to pay more for a larger net, 84% (or 245) were consistent with their choices (they also chose a larger net in scenario 18) and the remaining 16% (or 47) were inconsistent.

Based on these results the majority of individuals were rational and consistent. Nonetheless, the individuals whose answers were irrational or inconsistent with their survey answers were flagged so that the analysis could include or exclude them and determine the degree to which they influenced the findings. The results in the main text present the results without individuals who were irrational or inconsistent.

Table 2. Internal validity tests: participants' rational and consistent question tests

*Always-buyers Respondents:*

Another internal validity check was identifying respondents who always or nearly always choose one outcome or alternative with the best level of one attribute. This single attribute or outcome dominated-preference can bias model estimation ^2^. A tabulation of the total number of participants by how many times they chose to buy a net out of the 18 survey questions (see appendix Table 3) revealed that out of the 628 participants in the study 48% (or 303) chose to buy a net in all the 18 DCE survey questions.

Table 3. Always-Buyers

| Number of times (out of 18 choice questions) that participants chose to buy (either net A or net B) | Frequency | |
| --- | --- | --- |
|  | No. of  participants | Percent |
| 18 (Always chose to buy) | 303 | 48.25 |
| 17 | 73 | 11.62 |
| 16 | 44 | 7.01 |
| 15 | 48 | 7.64 |
| 14 | 45 | 7.17 |
| 13 | 35 | 5.57 |
| 12 | 31 | 4.94 |
| 11 | 17 | 2.71 |
| 10 | 14 | 2.23 |
| 9 | 3 | 0.48 |
| 8 | 7 | 1.11 |
| 7 | 6 | 0.96 |
| 6 | 0 | 0.00 |
| 5 | 1 | 0.16 |
| 4 | 0 | 0.00 |
| 3 | 0 | 0.00 |
| 2 | 0 | 0.00 |
| 1 | 0 | 0.00 |
| 0 (Always chose not to buy) | 1 | 0.16 |
| All | 628 | 100 |

We were initially skeptical that so many participants would be content purchasing a net in every single choice pair. It may be possible that these participants wanted a LLIN regardless of the attribute levels and thus were not fully engaged in providing information about their true preference. However, post-DCE survey interviews with all the survey interviewers revealed that the great majority of participants showed engagement in the experiment by asking questions and verifying that they understood the choice at hand for all the 18 questions and reassured that it was rare to have seen disengaged participants. Some analysts believe that real consumers apply the elements of anchoring to one outcome in their market behavior, so to avoid biasing results by excluding the always-buyers, these 303 respondents were kept in the analysis and their removal was tested in sensitivity analysis, see appendix Table 5.

## LLIN Purchase Probabilities by Price for Different LLIN types

Table 4a lists predicted probability of purchase (LLIN demand) by willingness-to-pay values for the range of prices tested in the DCE. These predicted probabilities were estimated using the same multivariate logistic regression with random effects described in the manuscript and the logistic regression coefficient values were converted to marginal effects to ease their interpretation as elasticities of the probability of purchase. To obtain all the data points in Tables 4a-b, margins were calculated for every 1 GHS increment in the price between the price values of 5 GHS and 65 GHS. In Table 4a, the left columns list data for the average type of LLIN tested in the DCE. In this case, the margins were set to predict purchase probabilities for the average type of LLIN tested in the DCE. The stata command used was: margins, at(priceGHS =(5(1)65)) atmeans vsquish post. The columns to the right list data for the most attractive LLIN. In this case, the margins were set to predict purchase probabilities for LLINs with the combination of preferred attribute levels (i.e. shape: circular; size: queen, and; entry design: zipper). The stata command used was: margins, at(priceGHS =(5(1)65) ShapeCir=1 SizeQ=1 EntryZipper=1) atmeans vsquish post.

Similarly, Table 4b lists the predicted price elasticities of demand by LLIN price for the range of prices tested in the DCE. The range of elasticities for the lowest and highest WTP values were -0.015 and -0.22, respectively. The stata command used was: margins, eyex(priceGHS) pr(pu0) atmeans vsquish post.

Table 4a. LLIN Average Probability of Demand by Price & LLIN Type

Table 4b. LLIN Price Elasticity of Demand by Price Point

## Invalid Respondents & Sensitivity Analysis of Regression Analysis

Before running the regression models, we ran estimates of the correlation between the demographic and socioeconomic variables to ensure that these were not multicollinear. The appendix Table 5a, listing the correlation coefficients, shows that these variables did not suffer from multicollinearty.

Table 5a. LLIN Price Elasticity of Demand by Price Point

|  | Demand | Female | Age Cat | Education | Married | Wealth Index | Rural | No. of HH members |
| --- | --- | --- | --- | --- | --- | --- | --- | --- |
| Demand | 1.00 |  |  |  |  |  |  |  |
| Female | 0.02 | 1.00 |  |  |  |  |  |  |
| Age Cat | -0.01 | 0.02 | 1.00 |  |  |  |  |  |
| Education | 0.00 | -0.02 | 0.03 | 1.00 |  |  |  |  |
| Married | 0.00 | 0.06 | 0.10 | -0.05 | 1.00 |  |  |  |
| Wealth Index | -0.01 | 0.01 | 0.03 | 0.15 | 0.01 | 1.00 |  |  |
| Rural | -0.02 | -0.12 | -0.01 | -0.17 | 0.01 | -0.01 | 1.00 |  |
| No. of HH members | 0.02 | 0.12 | 0.05 | -0.05 | 0.18 | 0.09 | -0.01 | 1.00 |

The appendix Table 5b, shows five columns A-E. Column A shows results for the full sample of 628 participants. Column B shows results for the full sample of 628 participants plus the use of dummy variables to control for the irrational, inconsistent and always-buyers instead of removing them. Column C shows results for the sub-sample of 541 participants excluding those with irrational and inconstant choice behavior (the same as the main study results). Column D is the same as column C expect that a dummy variable is included to control for the always-buyers. Results from all four models in columns A to D are robust (consistent and practically identical). Column E shows results for the sub-sample of 325 participants who did not anchor to always buy (i.e. analysis removed the always-buyers). Without the always-buyers, results show slightly different results for the shape attribute in which the rectangular 1-point-hang (R1p) shape is not statistically preferred to the 4-point-hang (R4p) shape, instead only the shape conical is preferred to the R4p. This means that including the always-buyers changes the results so that the R1p (like the conical) shape are both preferred to R4p.

Table 5b. Sensitivity Analysis of Regression analysis

Note: Presentation of the coefficients for sociodemographic variables was suppress to improve legibility of the table. These values are available upon request. *** is a value with a 99% confidence interval; ** is a value with a 95% confidence interval; * is a value with a 90% confidence interval

## Regression Analysis Stratification

Stratification of the analysis by sub-groups showed coefficients that were generally similar. One exception was finding statistically insignificant price effects for the Western region, but this may have been due to the small sample in that sub-group (143). Likewise, as expected, among the 378 individuals without a LLIN in the HH, the various attribute improvements were strongly associated (p-values<0.00) with increased demand for LLINs. But among the 163 respondents who had at least one LLIN in the HH, the attribute improvements were not statistically associated with demand, except for LLINs with size queen instead of double.

Similarly, for the 219 males, improvements to the shape attributes increased LLINs demand (p-values <00.0 or <0.05). Male respondents showed a particularly large preference for shape trade-offs for R4p vs. conical (6.25% p-value<0.00), but for the 322 females, shape improvements had no statistically significant effect on demand. For the 177 wealthiest, improvements to all attribute levels (expect R1p) increased demand (p-values <00.0 or <0.05), but for the 177 least wealthy, only improvements to shape R4p (p-value <0.1) and the zipper entry design (p-value<0.05) increased demand. Similarly, for Ashanti, but not in Greater Accra, entry design trade-offs to zippers increased demand buy as much as 6.97% (p-value<00). In Western, neither shape nor size showed an effect on demand but this is likely due to its small data sample. In Ashanti, shape R1p was preferred to both conical and R4p (p-value 0.00), but in Greater Accra, shape conical increased demand by as much as 8.27% (p-value <0.00). Results for the sub-group of rural participants were not listed because the sample size was smaller than the minimum of 100 respondents recommended for drawing statistical inferences ^2^.

Table 6. Regression analysis stratified by sup-populations

Note: P-value asterisks mean that the estimate on the change in the demand for LLINs is statistically different than the average demand for LLINs (43.8%): *** is a value with a 99% confidence interval; ** is a value with a 95% confidence interval; * is a value with a 90% confidence interval, and no asterisk means that the value is not statistically different than the mean demand. Results are based on a multivariate logistic regression with random effects controlling for respondents' socio-demographic characteristics, the other LLIN choice attributes, interviewer, and for participant exhaustion.

## Public cost savings and commercial market revenue

Total public cost for two scenarios:

Scenario A - The public sector buys one basic LLIN for each household regardless of income. In this scenario households receive publicly subsidized basic LLINs. The LLIN is purchased at the current average production cost.

Scenario B - The public sector buys one basic LLIN for each household regardless of income. But, in this scenario the private market sells LLINs with characteristics that make consumers be willing to buy these and only the households in poverty or those not willing to buy an enhanced LLIN with their own out of pocket spending receive publicly subsidized basic LLINs. Subsidized LLINs are purchased at the current average production cost and private market LLINs are purchased at the average willingness to pay (WTP) value.

Table 7a list the parameters, parameters values and sources of the data used in the simulation model. The data on the total number of households living in the 3 study areas (e.g. districts with lower than the average poverty rate, 0.7%-9.6%) is listed by district in Table 7b.

We use two different simulation models to compare the public sector savings between policy scenarios A and B. The first simulation (Figure 1a) assumes that we are estimating the public cost for buying LLINs to meet current coverage levels of HHs with at least 1 LLIN per HH. The second simulation (Figure 1b) assumes that we are estimating the public cost for buying LLINs for HHs without any LLINs. Thus, this second simulation models the additional cost and savings needed to close the LLIN ownership gap of HHs with at least 1 LLIN per HH. Additionally, Figure 1aX replicates estimates from Figure 1a but changing the units of estimation from the cost to the total population in the study area to the cost per every 100 HHs in the study area. We run 1,000,000 iterations of the model in a Monte Carlo simulation to produce confidence intervals around cost and savings estimates.

Results: Both simulation models show that the public sector outlay would be reduced by 39% (95% C.I. 50% and %28%). The simulation in Figure 1a also shows that the private market could increase current coverage levels by 85% among non-poor households in the study area.

Table 7a. Model Parameters

Table 7b. Poverty estimates by district

Figure 1a. Policy Analysis Model – Estimates for all 2.6 Million Households in the Study Areas (Estimates Cost to Meet Current Coverage Levels)

† Districts with poverty rate (0.7%-9.6%) lower than the average (11.6%)

*Assumes conservative scenario that all households in poverty are not able to buy a LLIN OOP

LLIN: Long-Lasting Insecticidal Net; HH: Household; OOP: Out-of-pocket; WTP: Willingness to Pay

Table 7c. Summary of Key Results, Policy Analysis Model – Estimates for all 2.6 Million Households in the Study Areas (Estimates Cost to Meet Current Coverage Levels)

Figure 1b. Policy Analysis Model – Estimates for all 2.6 Million Households in the Study Areas (Estimates Additional Cost to Close the Current Coverage Level Gap)

† Districts with poverty rate (0.7%-9.6%) lower than the average (11.6%)

*Assumes conservative scenario that all households in poverty are not able to buy a LLIN OOP

LLIN: Long-Lasting Insecticidal Net; HH: Household; OOP: Out-of-pocket; WTP: Willingness to Pay

Figure 1c. Policy Analysis Model – Estimates for a Random Sample of 100 Households in the Study Areas

† Districts with poverty rate (0.7%-9.6%) lower than the average (11.6%)

*Assumes conservative scenario that all households in poverty are not able to buy a LLIN OOP

LLIN: Long-Lasting Insecticidal Net; HH: Household; OOP: Out-of-pocket; WTP: Willingness to Pay

## References

1. Ghana Statistical Service (GSS) *Ghana Malaria Indicator Survey 2016 Sampling.* World Bank;2017.

2. Bridges JFP, Hauber AB, Marshall D, et al. Conjoint Analysis Applications in Health—a Checklist: A Report of the ISPOR Good Research Practices for Conjoint Analysis Task Force. *Value in Health.* 2011;14(4):403-413.

1. Mensah E, Piccinini D, Osei T, Dontoh A, Kim S, Alfonso YN. Catalyzing the Commercial Market for LLINs in Ghana, A Market Analysis. URIKA Research and PSMP project; 2018. <https://www.privatesectormalaria.org/2018/10/24/catalyzing-the-commercial-market-for-llins-in-ghana-a-market-analysis-2018/> [↑](#footnote-ref-1)
2. ibid [↑](#footnote-ref-2)
